# Association between sitting time at work and the onset of major depressive episode: a 1-year prospective cohort study using the Bayesian regression

**DOI:** 10.1186/s12889-021-12059-y

**Published:** 2021-10-29

**Authors:** Kazuhiro Watanabe, Norito Kawakami

**Affiliations:** 1 Department of Public Health, Kitasato University School of Medicine, 1-15-1 Kitazato, Minami-ku, Sagamihara, 252-0374 Japan; 2 Department of Mental Health, Graduate School of Medicine, The University of Tokyo, 7-3-1 Hongo, Bunkyo-ku, Tokyo, 113-0033 Japan

**Keywords:** Sedentary, Physical activity, Depressive disorder, Workers, Bayesian analysis

## Abstract

**Background:**

Although sedentary behavior is associated with the onset of major depressive disorder, it remains unclear whether sedentary behavior at work increases the risk of depression. The present study used the Bayesian approach to investigate the association between sitting time at work and the onset of major depressive episode (MDE).

**Methods:**

A 1-year prospective cohort study was conducted among 233 Japanese workers without MDE (response rate: 4.3%). MDE onset was assessed using the self-reported WHO Composite International Diagnostic Interview version 3.0. A Bayesian Cox proportional hazard model was used to estimate the hazard ratio (HR) between long sitting time at work and MDE onset.

**Results:**

A total of 231 workers were included in the analysis. During the follow-up, 1621 person-months were observed, and six participants experienced MDE onset. Incident rates per months were 0.34, 0.11, and 1.02% in short (< 7.2 h per day), medium (7.2–9.5 h), and long (9.5+ h) sitting time at work, respectively. The estimated median posterior probability distribution of the HR of long sitting time was 3.00 (95% highest density interval [HDI]: 0.73–12.03). The estimated median remained positive after adjustment for physical activity level and other covariates (HR = 2.11, 95% HDI: 0.42–10.22). The 10-base Bayesian factor for H1 (HR = 1.00) compared with the alternatives (H0, HR = 1.00) was 0.68 in the adjusted model. The analysis, which treated sitting time at work as a continuous variable, estimated that the median of the posterior probability distribution of the HR of sitting time was 0.79 (95% HDI: 0.58–1.07. The 10-base Bayesian factor was 2.73 in the linear association.

**Conclusions:**

Long sitting time at work (9.5+ h per day) might be associated with MDE onset among workers. However, the linear association indicated conflicting results. Non-linear associations between sitting time and MDE onset might explain this inconsistency. The evidence for an adverse association between sitting time at work and MDE onset remains inconclusive.

## Background

Sedentary behavior is defined as any waking behavior involving an energy expenditure of 1.5 metabolic equivalents or less while in a sitting or reclining posture [1]. Such behavior has been associated with morbidity and mortality from various non-communicable diseases, even independent of physical inactivity [2, 3]. In addition, sedentary behavior is associated with an increase in the risk of mental disorders. A systematic review and meta-analysis [4] of 24 studies showed that the pooled relative risk (RR) of depression associated with sedentary behavior was 1.25 (95% confidence interval [CI]: 1.16–1.35). The association was still significant when only 11 longitudinal studies were considered (RR = 1.14, 95% CI: 1.06–1.21). More recently, Hallgren et al. [5, 6] conducted a 13-year prospective cohort study among Swedish adults with no mental disorder and found that mentally passive sedentary behaviors (e.g., TV watching, listening to music, sitting in the bathtub) were associated with a high risk of major depressive disorder (MDD).

In the economically active population, sedentary behavior at work is particularly prevalent [7], especially because modern society has seen a substantial and rapid increase in the proportion of workers whose occupations involve low physical activity and sedentary behavior [7, 8]. Descriptive studies have reported that the average sitting time at work ranges from 3.75 to 6.40 h per day, with white-collar workers unsurprisingly engaging in more sedentary behaviors than the blue-collar workers [9–12]. MDD is also prevalent and the most common mental disorder among Japanese workers, with the 12-month prevalence of MDD reported as 2.6% in the World Health Organization Mental Health Japan Survey [13].

Despite this, only a few prospective studies have investigated the role of sedentary behavior at work in the development of MDDs; previous studies targeting workers have focused on sedentary behaviors outside of work, such as TV viewing [14]. In addition, it remains unclear whether sedentary behavior at work exacerbates depression in the working population. Several prospective studies involving nurses and community residents have indicated a positive association between sedentary behavior and depression, with most of the sedentary behaviors taking place at work [15]. In another study, sedentary behaviors were negatively associated with the onset of MDD, if they were mentally “active” behaviors and not mentally “passive,” e.g., office work, sitting in a meeting, (Hazard Ratio [HR] = 0.74, 95% CI: 0.58–0.94) [6].

However, none of the above studies specifically investigated the working population, with many non-workers being included in their samples. Moreover, the studies did not distinguish sitting time at work from other sedentary behaviors. Occupational sitting would have a different association than sitting during home because domain-specific health-related behaviors have different relationships with depressive symptoms [16]. Furthermore, they did not adjust for important covariates in the association between sitting time at work and depression, such as working hours or job stressors. Therefore, the evidence remains inconclusive, and the impact of sedentary behaviors at work on depression should be specifically investigated in the working population.

In the present study, we aimed to investigate prospective associations between sedentary behaviors at work (sitting time during work) and the onset of major depressive episode (MDE) over a 1-year period, based on the standard diagnostic criteria detailed in the Diagnostic and Statistical Manual of Mental Disorders (DSM)-IV/V. We also investigated whether the associations were independent of total physical activity levels and job stressors (job demands, job control, and supervisor and coworker support). We hypothesized that long sitting time at work is associated with a higher risk of MDE, independent of physical activity levels and other critical occupational factors (e.g., job stressors).

To estimate the association, we adopted the Bayesian approach, given that the sample size was rather small. This approach provides a reasonable estimate, even when the sample size is smaller than that calculated using the null hypothesis significance testing (NHST) approach, because it considers prior probability distributions and the likelihood function [17].

## Methods

### Study design and setting

This was a 1-year prospective cohort study. Participants were recruited from three joint-stock companies (two manufacturing companies and one transportation company) in Japan between February and March 2019. The number of potential participants who worked in the three companies was 5750. After screening the participants based on the exclusion criteria (with MDE or officially absent due to mental health problems in the past 12 months), we obtained baseline information of the participants on the levels of sitting time at work, physical activities, and other covariates, as well as identifiers including their names and email addresses. They were followed up for 1 year to assess the onset of a major depressive episode (MDE). During follow-up, the participants received e-mails asking them to complete online surveys at three stages, 3-month (June 2019), 6-month (September 2019), and 12-month (March 2020). The research ethics committee of the Graduate School of Medicine and Faculty of Medicine, The University of Tokyo, Japan, approved the study protocol (2018054NI).

### Participants

**Fig. 1:**
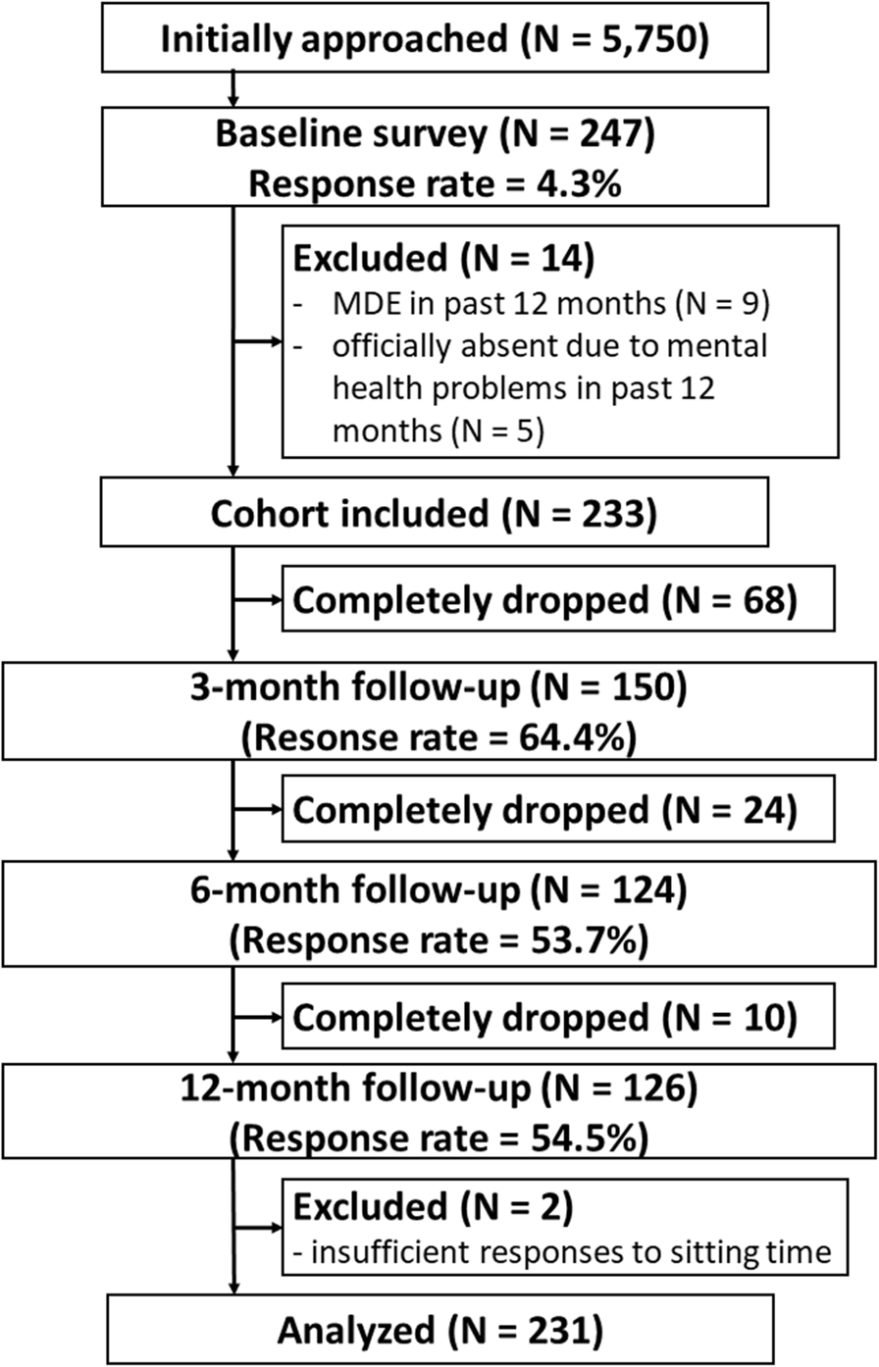
Study flowchart

Workers employed by one of the three companies were recruited for the study and intimated via e-mail invitations. They were asked to complete a baseline online survey. The first page of the online self-report questionnaire explained the terms and conditions of the study. If the participants agreed to the study and pressed the “agree” button on that page, they were granted access to further continue with the questionnaire. The eligibility criteria of the participants were as follows: [1] employee status, [2] age ≥ 20 years, and [3] comprehension of the questionnaires in Japanese. Participants were excluded if they [1] had suffered an MDE in the 12 months before the baseline survey or [2] had been officially absent due to mental health problems in the 12 months before baseline. Figure 1 shows the flowchart of the study. From the three companies, a total of 247 workers agreed to the terms and conditions of the study and completed the baseline survey (response rate: 4.3%). However, 14 workers were found to be ineligible for the study and were excluded; hence, 233 participants finally participated in the study. Among these, 102 (43.8%) participants dropped out during follow-up. The response rates of the follow-up surveys ranged from 53.7–64.4%. Two participants were further excluded from the analysis because they reported contradictory responses about the times when they arrived and left the workplace. Ultimately, 231 participants were included in the analyses.

### Measurement

Sitting time at work, MDE onset, and other variables were measured using the online self-reported questionnaire four times: at baseline and at the 3-month, 6-month, and 12-month follow-ups.

#### Onset of major depressive episode (MDE)

As the primary outcome, the onset of MDE during follow-up was assessed using the self-reported World Health Organization (WHO) Composite International Diagnostic Interview version 3.0 (CIDI 3.0) [18, 19]. The WHO-CIDI is a self-report questionnaire that was compiled according to the DSM-IV-TR. The validity of the Japanese version of the WHO-CIDI has already been proven among Japanese workers with an objective diagnosis; it was shown to have moderate sensitivity (71.4%) and high specificity (100.0%) [20]. The test-retest reliability of the measurement was reasonable (Gwet’s AC1 [21] = 0.93 and Yule’s Q [22] = 0.82), while the stability among positive cases was relatively low. In the follow-up surveys, the WHO-CIDI assessed whether the participants had experienced an onset of MDE since baseline. The baseline survey also utilized the questionnaire to exclude ineligible participants who had suffered from MDE in the 12 months before baseline.

#### Sitting time at work

Sitting time at work per day was measured using the Worker’s Living Activity-time Questionnaire (JNIOSH-WLAQ) [23], which calculates sitting time at work using a proportion method. The questionnaire first asked the participants how long they spent working per day. Next, it asked about the proportion of their time (0–100%) spent [1] sitting and [2] standing/walking during work, excluding commuting time. We calculated their sitting time at work by multiplying their time at the workplace and the proportion of sitting time. The reliability and validity of the JNIOSH-WLAQ have been proven in a previous study, with intra-class correlation coefficients of 0.72–0.98 and Spearman’s ρ with accelerometer = 0.67 [23]. We categorized the participants into three sitting time groups based on quartiles: short (< 25%, Q_1_ = 7.20 h), medium (25–75%, median = 8.36 h), and long (> 25%, Q_4_ = 9.53 h). In addition, sitting time as a continuous variable was also used in the sensitivity analysis.

#### Physical activity

Physical activity was measured using the Global Physical Activity Questionnaire (GPAQ v2) [24], which assesses overall levels of physical activity in 15 items asking about intensity (metabolic equivalents [METs]) and duration of activities in three domains (work, travel to and from places, and recreation). This scale is widely used and has demonstrated acceptable reliability and convergent validity among nine countries, including Japan [25]. We categorized the participants into three levels of physical activity (low, moderate, and high) according to the analysis guide of the GPAQ [26]. The high level of physical activity included ≥3 days vigorous-intensity activity with ≥1500 MET-minutes/week OR ≥ 7 days of any combination of walking or moderate- or vigorous-intensity activities (MVPA) with 3000 MET-minutes/week. The moderate level included ≥3 days of vigorous-intensity activity with ≥20 min/day OR ≥ 5 days of moderate-intensity activity or walking with ≥30 min/day OR ≥ 5 days of any combination of walking or MVPA with ≥600 MET-minutes/week. The low level refers to not meeting the criteria of either high or moderate.

#### Job stressors

Four types of job stressors (job demands, job control, and supervisor and coworker support) were measured using the subscales of the Brief Job Stress Questionnaire (BJSQ) [27], which has three items on each subscale, all of which are rated on a four-point Likert scale (for job demands and job control: 1 = not at all, 4 = very much so; for supervisor and coworker support: 1 = not at all, 4 = extremely). In the present study, the total scores of the subscales were used, with higher scores denoting higher job demands, job control, or supervisor and coworker support. Cronbach’s α of the four subscales ranged from 0.652 to 0.823 in the baseline survey.

#### Covariates

The self-reported demographic variables used in modeling included sex (men [reference] and women), age (20–39 [reference], 40–49, 50–59, and 60+ years), educational status (≤ 12 years [reference], 13–15 years, and 16+ years), marital status (married and unmarried), and household income (low [< 5 million yen, reference], medium [5–10 million yen], and high [10+ million yen]). Drinking (never [reference], rarely, sometimes, almost daily) and smoking (never smoking [reference], smoked before and quit, and currently smoking) were also measured as other lifestyle factors. Moreover, working hours per week (< 40 h [reference], 40–45 h, 46–60 h, and 60+ h) were measured as an important confounder of sitting time at work.

### Sample size calculation

The sample size was not based on a calculation because the present study did not adopt the NHST approach. As a reference, if we wanted to detect the association between sitting time at work and MDE onset, a large number of participants (65,310, with 1829 new MDE-positive cases) would be needed with a hazard ratio (HR) of 1.14 [4], a 1-year cumulative incidence rate of 0.028 [20], an α-error probability of 0.05, and a statistical power (1-β) of 0.80, as calculated using the *power cox* command in Stata version 16 [28].

### Statistical analysis using the Bayesian approach

#### Strength of the Bayesian approach in the study

Instead of the NHST approach, we used the Bayesian approach to estimate the HR for the association between long sitting time at work and MDE onset. This approach estimates the posterior probability distribution of the parameters of the model based on the prior probability distribution and likelihood function. Given the small sample size and high dropout rate in the present study, the framework of the Bayesian approach is more robust than that of the NHST approach. As described above, if we had adopted the NHST approach, a large number of participants would have been needed to conclude whether the hypothesis was supported according to *p*-values. The Bayesian approach relies less on point estimates and significance levels, and instead utilizes prior distributions that can integrate previous findings. This helps stabilize and anchor parameter estimates in cases of small sample sizes [17].

#### Statistical modeling

Characteristics of the participants at baseline were summarized based on those who completed and dropped out of the follow-up surveys. We calculated the total person-months observation, the number of new MDE cases during the 1-year follow-up, and incident rates stratified by sitting time at work (short, medium, and long). Kaplan–Meier curves were also depicted for each group. The Cox proportional hazard model was used as a parametric survival model to estimate the HR for the association between long sitting time at work and MDE onset. The posterior distribution of the crude HR was estimated, with participants who had a short sitting time used as a reference group. As the main analysis, we estimated the posterior distribution of the HR between long sitting time (short and medium [reference] vs. long) at work and MDE onset. We adjusted the level of physical activity (low [reference], moderate, and high) and other covariates. Based on the posterior distribution, the point of median and 95% high-density interval (HDI) were reported. A propensity score was created and used to adjust the covariates and reduce complexity. The propensity score was estimated using a logistic regression model, with the groups of sitting time at work as the dependent variable [(short + medium) and long] and the covariates as independent variables, including sex, age, educational status, marital status, household income, drinking and smoking behavior, and working hours. Additionally, we conducted a sensitivity analysis to estimate the dose-response relationship between the long sitting time at work and MDE onset, treating sitting time at work (hours) as a continuous variable.

The models were fitted to an adaptive Metropolis–Hastings Markov chain Monte Carlo (MCMC) method, which is provided by the *Bayes:streg* command in Stata version 16 [28]. In the simulation, a total of three chains of 20,000 samplings from the posterior probability distribution were obtained. The first half of the simulation (10,000 in each chain) consisted of burn-in sampling and was excluded from the estimation. To reduce autocorrelation, sampling was performed every two draws. To check the convergence of the MCMC samplings, histograms, traces of the samplings, and autocorrelations were visually plotted among the three chains. The maximum Gelman–Rubin Rc value [29, 30] was also calculated, which is widely used as the convergence diagnostic value among multiple chains; an Rc < 1.1 denotes good convergence.

#### Prior probability distribution

To estimate the parameters in a stable manner, we used the normal distribution *N* ~ (0, 3.5) as the coefficients (logarithm of HRs) of long sitting time at work, the propensity score, and the base hazard of MDE onset. The standard deviation of the distribution (3.5) indicated that the point of HR at ±1 standard deviation (SD) was EXP (− 3.5) = 0.03, and EXP (3.5) = 33.11. We also used the normal distribution *N* ~ (− 1.86, 1.0) as the coefficients of the physical activity level. The mean of the distribution (− 1.86, EXP [− 1.86] = 0.83) was derived from a previous meta-analysis [31] indicating that sufficient physical activity could reduce the risk of depression by 17% (RR = 0.83, 95% CI, 0.79–0.88). No other parameters had prior distributions.

#### Hypothesis testing

As a statistical value for our hypothesis testing, the base-10 logarithm of Bayes factor (log_10_BF_10_) was used [32]. The Bayes factor is calculated based on the ratio of marginal likelihoods of the two models, incorporating information about prior models. This value quantifies the evidence for our hypothesis (H1, HR = 1.00) compared with the alternatives (H0, HR = 1.00). Jeffreys [32] stated that evidence for the hypothesis was insufficient when log_10_BF_10_ ≤ 0.5, substantial when log_10_BF_10_ ≤ 1, strong when log_10_BF_10_ ≤ 2, and decisive when log_10_BF_10_ > 2.

## Results

### Characteristics of the participants

Participants’ characteristics are presented in Table 1. Approximately three-quarters of the participants were men, and more than half had received 16 or more years of education. The medium household income per year was 5–10 million yen, and 55 (25.1%) participants earned more than 10 million yen per year. With regard to physical activity, most participants (57.1%) reported low levels, while a quarter reported high levels. In the JNIOSH-WLAQ assessment, the average time was 10.73 h (SD = 1.32 h) from arrival until leaving each day. During their work, the participants were sitting for 8.22 h per day on average (SD = 2.08 h). The quartiles of sitting time at work were 7.20 (25%), 8.36 (median), and 9.53 (75%) hours. Except for the primary outcome, no significant difference was found between the participants who completed and dropped out of the follow-up surveys. The participants who dropped out during the follow-up reported shorter sitting time at work compared with those who completed the follow-up.

**Table 1:** Characteristics of the participants at baseline (*N* = 231)

	Total (*N* = 231)	Follow-up completers (*N* = 130)	Dropout during follow-up (*N* = 101)	Difference (*p*-value)
	N (%) Mean (SD)	N (%) Mean (SD)	N (%) Mean (SD)	
Sex				0.881
Men	175 (75.8)	98 (75.4)	77 (76.2)	
Women	56 (24.2)	32 (24.6)	24 (23.8)	
Age				0.425
20–39	68 (29.4)	37 (28.5)	31 (30.7)	
40–49	60 (26.0)	33 (25.4)	27 (26.7)	
50–59	94 (40.7)	57 (43.8)	37 (36.6)	
60+	9 (3.9)	3 (2.3)	6 (5.9)	
Educational status (year)				0.149
≤ 12	70 (30.3)	33 (25.4)	37 (36.6)	
13–15	29 (12.6)	19 (14.6)	10 (9.9)	
16+	132 (57.1)	78 (60.0)	54 (53.5)	
Marital status				0.255
Married	171 (74.0)	100 (76.9)	71 (70.3)	
Not married	60 (26.0)	30 (23.1)	30 (29.7)	
Household income per year				0.123
Low (< 5 million yen)	53 (22.9)	24 (18.5)	29 (28.7)	
Medium (5–10 million yen)	123 (53.2)	76 (58.5)	47 (46.5)	
High (10+ million yen)	55 (23.8)	30 (23.1)	25 (24.8)	
Physical activity				0.870
Low	132 (57.1)	76 (58.5)	56 (55.4)	
Moderate	41 (17.7)	23 (17.7)	18 (17.8)	
High	58 (25.1)	31 (23.8)	27 (26.7)	
Drinking				0.418
Never	36 (15.6)	20 (15.4)	16 (15.8)	
Rarely	43 (18.6)	22 (16.9)	21 (20.8)	
Sometimes	89 (38.5)	56 (43.1)	33 (32.7)	
Almost daily	63 (27.3)	32 (24.6)	31 (30.7)	
Smoking				0.074
Not smoking	135 (58.4)	83 (63.8)	52 (51.5)	
Smoked before and quitted	55 (23.8)	30 (23.1)	25 (24.8)	
Currently smoking	41 (17.7)	17 (13.1)	24 (23.8)	
Job stressors				
Job demands	M = 8.32 (SD = 2.03)	M = 8.27 (SD = 1.88)	M = 8.39 (SD = 2.22)	0.070
Job control	M = 8.68 (SD = 1.61)	M = 8.85 (SD = 1.64)	M = 8.46 (SD = 1.55)	0.704
Supervisor support	M = 7.80 (SD = 2.15)	M = 7.81 (SD = 2.20)	M = 7.79 (SD = 2.11)	0.524
Coworker support	M = 8.07 (SD = 1.97)	M = 8.15 (SD = 1.97)	M = 7.97 (SD = 1.97)	0.589
Working hours (per week)				0.083
< 40 h	34 (14.7)	18 (13.8)	16 (15.8)	
40–45 h	77 (33.3)	50 (38.5)	27 (26.7)	
46–60 h	104 (45.0)	57 (43.8)	47 (46.5)	
60+ h	16 (6.9)	5 (3.8)	11 (10.9)	
Sitting time at work (hours per day)	M = 8.21 (SD = 2.08)	M = 8.34 (SD = 1.89)	M = 8.06 (SD = 2.29)	0.020

### Sitting time at work and MDE onset

**Fig. 2:**
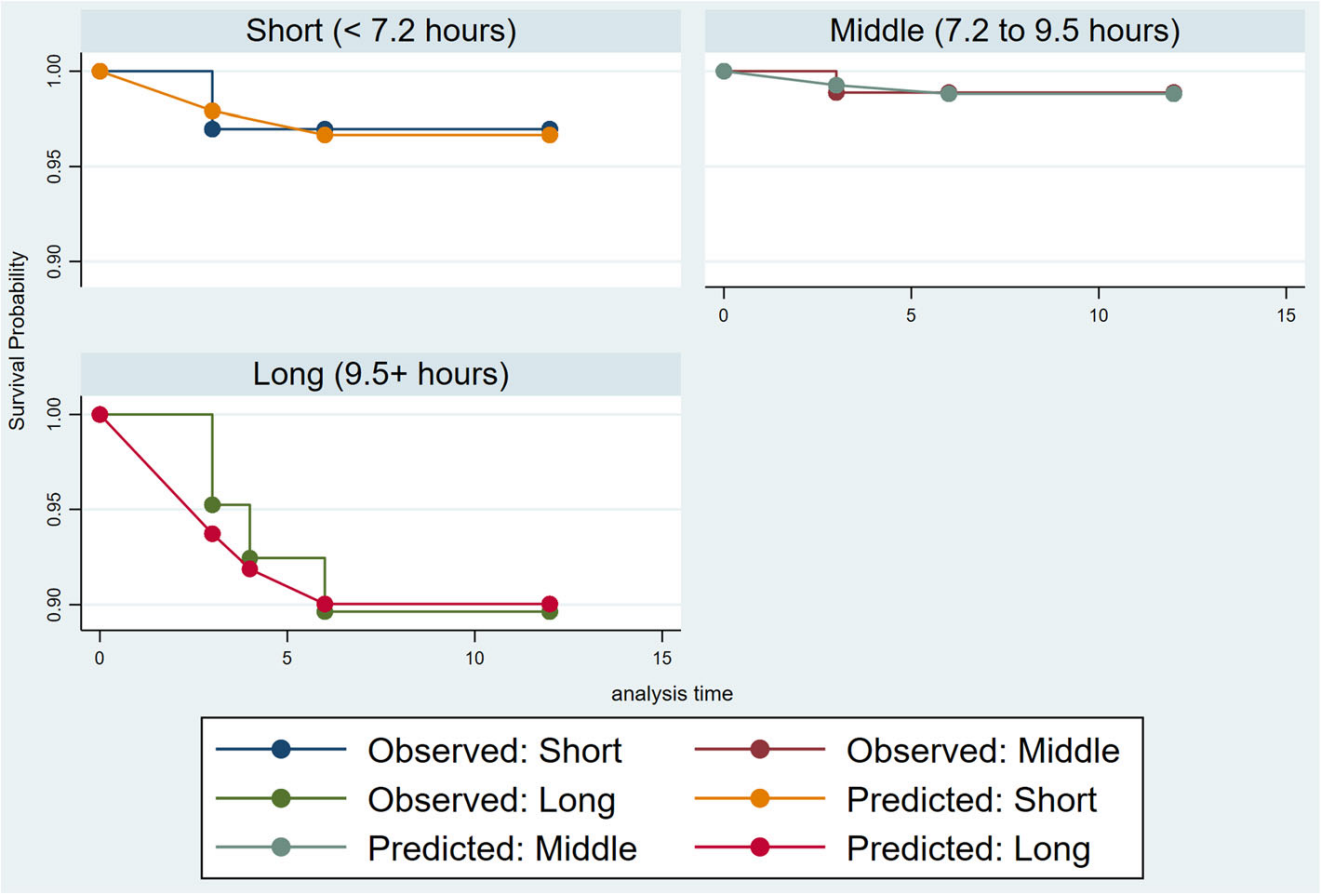
Kaplan–Meier curves stratified by the three groups classified according to sitting time at work (per day)

Table 2 shows the incident rates and HRs of the onset of MDE stratified by sitting time at work. During the 1-year follow-up, 1621 person-months were observed, and a total of six participants experienced MDE onset. The occupations of these six participants were managerial (*n* = 2), professional/technical (*n* = 1), clerical (*n* = 2), and others (*n* = 1). The incidence rate of MDE was 0.37% per month, while the cumulative incidence rate was 2.6%. In both the short sitting time group (< 7.2 h, *n* = 58) and the medium group (7.2–9.5 h, *n* = 115), one participant experienced MDE onset in each group. In contrast, in the long-sitting group, four participants experienced MDE. The estimated medians of the posterior probability distributions of the HRs were 0.23 (95% HDI: 0.03–1.36) in the medium group and 1.54 (95% HDI: 0.35–7.52) in the long group according to the Bayesian Cox proportional hazard model. The maximum Gelman–Rubin Rc value was 1.006, which indicates sufficient convergence among the three chains of MCMC samplings. The Kaplan–Meier curve (Fig. 2) showed a relatively steep decline in survival probability in the long sitting group.

**Table 2:** Person-months observation, cases, and incident rates for the association between sitting time at work and MDE onset (*N* = 231)

	N (%)	Person-months observed	Case (N)	Incident rate (per month)	HR Median	95% HDI		Post prob. (HR > 1.00)
						low	high	
Sitting time at work (per day)								
Short (< 7.2 h)	58 (25.1)	294	1	0.0034	1.00	–	–	–
Medium (7.2–9.5 h)	115 (49.8)	933	1	0.0011	0.23	0.03	1.36	0.051
Long (9.5+ h)	58 (25.1)	394	4	0.0102	1.54	0.35	7.52	0.716
Total	231	1621	6	0.0037

**Fig. 3:**
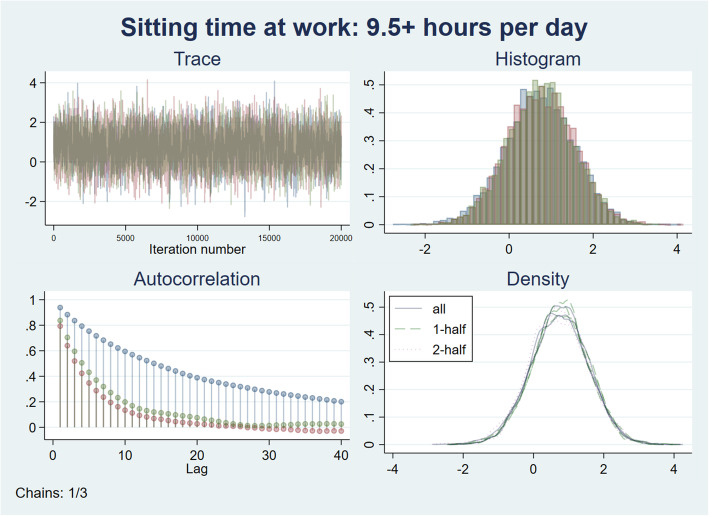
Posterior probability distributions, trace plot, and autocorrelations for the coefficient of long sitting time at work in the Cox proportional hazard model

In the main analysis, Table 3 shows the results of the Bayesian Cox proportional hazard model to estimate the HR between long sitting time at work (9.5+ h per day) and MDE onset. In a crude model, the estimated median of the posterior probability distribution of the HRs was 3.00 (95% HDI: 0.73–12.03). The posterior probability of HR > 1.00 was 93.9%. The estimation of positive association remained after adjustment for the level of physical activity (HR = 2.93, 95% HDI: 0.73–11.89) and the other covariates (HR = 2.11, 95% HDI: 0.42–10.22). Figure 3 shows the posterior probability distribution, trace, and autocorrelations of the three chains of MCMC samplings in the fully adjusted model. The posterior probability of HR > 1.00 was 82.1%. The log_10_BF_10_ for H1 (HR = 1.00) compared with the alternatives (H0, HR = 1.00) were 1.55, 1.40, and 0.68, respectively. These values indicate strong evidence for H1 in the crude and physical activity-adjusted model and substantial evidence for H1 in the fully adjusted model [32].

**Table 3:** Cox proportional hazard model to estimate the hazard ratio (HR) between long sitting time at work (9.5+ h) and MDE onset (*N* = 231)

	Crude model				Adjusted model 1^†^				Adjusted model 2^‡^			
	HR (Median)	95% HDI		Post prob. (HR > 1.00)	HR (Median)	95% HDI		Post-prob (HR > 1.00)	HR (Median)	95% HDI		Post-prob (HR > 1.00)
		low	high			low	high			low	high	
Sitting time at work												
< 9.5 h/day (ref)	1.00	–	–	–	1.00	–	–	–	1.00	–	–	–
9.5+ h/day	3.00	0.73	12.03	0.939	2.93	0.73	11.89	0.935	2.11	0.42	10.22	0.821
Physical activity (ref: low)												
Moderate					1.13	0.28	3.96		1.09	0.28	3.76	
High					0.65	0.14	2.34		0.67	0.15	2.57	
Propensity score									3.01	0.17	49.22	
Gelman–Rubin Rc (max)	1.002				1.003				1.021			
DIC	65.98				67.25				67.40			
Log marginal-likelihood	−43.10				−43.46				−41.24			
log_10_BF_10_	1.55				1.40				0.68			

Note. *HDI* highest density interval, *DIC* deviance information criterion, *log*_*10*_*BF*_*10*_ log Bayes factor compared to H0 (HR = 1.00). †Adjusted for physical activity levels. ‡Adjusted for physical activity levels and propensity scores The propensity score was created based on sex, age, educational status, marital status, household income, job stressors (job demands, job control, supervisor and coworker support), and working hours

The sensitivity analysis which treated sitting time at work as a continuous variable (Table 4) indicated that the estimated median of the posterior probability distribution of the HRs were 0.90 (95% HDI: 0.69–1.21) in the crude model, 0.91 (95% HDI: 0.69–1.22) in the physical activity level adjusted model, and 0.79 (95% HDI: 0.58–1.07) in the fully adjusted model. The posterior probability of HR > 1.00 in the fully adjusted model was 6.2%, and the log_10_BF_10_ for H1 (HR = 1.00) compared with the alternatives (H0, HR = 1.00) was 2.73.

**Table 4:** Cox proportional hazard model to estimate the hazard ratio (HR) between sitting time at work and MDE onset (*N* = 231)

	Crude model				Adjusted model 1^†^				Adjusted model 2^‡^			
	HR (Median)	95% HDI		Post prob. (HR > 1.00)	HR (Median)	95% HDI		Post prob. (HR > 1.00)	HR (Median)	95% HDI		Post prob. (HR > 1.00)
		low	high			low	high			low	high	
Sitting time at work per day	0.90	0.69	1.21	0.242	0.91	0.69	1.22	0.257	0.79	0.58	1.07	0.062
Physical activity (ref: low)												
Moderate					1.18	0.28	4.11		1.11	0.27	3.87	
High					0.66	0.14	2.46		0.74	0.16	2.88	
Propensity score									18.95	0.92	365.64	
Gelman-Rubin Rc (max)	1.001				1.002				1.011			
DIC	70.84				71.86				68.93			
Log marginal-likelihood	−44.94				−45.29				−39.19			
log_10_BF_10_	−0.29				−0.43				2.73			

Note. *HDI* highest density interval, *DIC* deviance information criterion, *log*_*10*_*BF*_*10*_ log Bayes factor compared to H0 (HR = 1.00). †Adjusted for physical activity levels. ‡Adjusted for physical activity levels and propensity scores The propensity score was created based on sex, age, educational status, marital status, household income, job stressors (job demands, job control, supervisor and coworker support), and working hours

## Discussion

The results indicated that a long sitting time at work (9.5+ h per day) was associated with an approximately 2-fold higher risk of MDE onset, with a moderate to high probability (Prob HR > 1.00 = 82.1%) and with substantial evidence (log_10_BF_10_ for H1 = 0.68). The estimated median remained positive even after adjusting for the level of physical activity, job stressors, and other covariates. However, the sensitivity analysis did not support a linear positive association, indicating the inverse (protective) results with decisive evidence (log_10_BF_10_ for H1 = 2.73). Although the linear association between sitting time at work and MDE onset is unclear, sitting at work for 9.5 h or longer might be positively associated with MDE onset among workers. This was the first prospective study to examine the association between sitting time at work and the onset of MDE among workers. The findings of the study could be useful in motivating the working population to reduce the prolonged hours of sitting at work and to prevent the incidence of MDD.

The key finding of the study was that extremely long sedentary behavior at work had an adverse association with MDE onset among workers. This is consistent with previous studies targeting the working population [14, 15]. However, it should be noted that a linear negative association was found between the continuous variable of sitting time at work and the risk of MDE, contrary to the key finding and our hypothesis. A possible explanation for this discrepancy may be that there is a unique group at high risk of MDE onset amongst extremely long sitting time at work, while medium sitting time at work may be associated with a lower risk of MDE. Such a non-linear association between sitting time and MDE onset may explain these results. Another possible explanation for the linear negative association between sitting time and MDE is that the results might be confounded by the mentally active behavior of the office work. Hallgren et al. [5] reported that mentally active sedentary behaviors (including office work) were protective against MDD onset. Not sitting time, but hours of mentally active work may be protective for MDE onset. Our results could be replicated in future research by testing these hypotheses.

The underlying mechanisms of this positive association have been discussed in previous studies [6, 33–38]. Reduced physical activity and social withdrawal caused by low levels of activity and long sitting are some examples of a potential mechanism [33]. However, the present study found that the positive association may still occur regardless of physical activity level, so other explanations are needed. Recently, Hallgren et al. [6] suggested that sleep problems, such as insomnia and sleep disturbance, may mediate this mechanism. Sleep problems are both an important risk factor and a symptom of MDD [34], and sedentary behavior is a proven risk factor for sleep problems [35]. Interestingly, one multinational, cross-sectional study found that the positive association between sleep problems and sedentary behavior was independent of physical activity, depression, obesity, or physical diseases [36]. The authors suggested that exposure to light-emitting diodes during sitting time might be detrimental to circadian rhythms and sleep cycles [37]. This explanation is consistent with the present study results, which showed that the four cases of MDE in the long sitting group had occupations that involved heavy computer use (i.e., managerial and professional/technical) until night (19:00 to 23:00). Another potential explanation for this association could be reduced cognitive function. A recent meta-review [38] indicated that higher levels of sedentary behavior are associated with reduced cognitive performance in adults. Another important aspect is patterns of sitting, such as bouts or breaks, which could not be measured in the present study. The meta-review concluded that breaking up sitting time may benefit body composition and markers of cardiometabolic risk [38]. The association of break of sedentary behaviors with MDE onset is unknown, so future studies are needed to test the association.

### Strengths and limitations of the study

This study adopted the Bayesian approach instead of the NHST approach. Given the small sample size and high dropout rate in the present study, the framework of the Bayesian approach could make us estimate more robust associations than that of the NHST approach.

However, this study has several limitations. First, the categorization (short, medium, and long) and dichotomization ((short + medium) and long] of sitting time at work were not registered as the protocol and arbitrarily defined after the survey. A propensity score was also created based on dichotomization. These operations were biased according to the authors’ hypotheses. Indeed, sensitivity analysis did not support adverse associations between sitting time and MDE onset. Second, the low response rates of the baseline (4.3%) and the follow-up surveys (53.7–64.4%) may have caused selection and attrition bias, respectively. In the present study, workers who dropped out reported shorter sitting time at work compared with those who completed the follow-up. Workers who had a long sitting time and were depressed may have been reluctant to participate in the study, so we may have underestimated the association. Third, all variables were measured using self-reported questionnaires, which may provide biased information and measurement errors. Fourth, several potential confounders of the association between sitting time and MDE onset were not adjusted for in the present study. For instance, we did not measure and thus could not include sleep problems in the statistical model, which are currently the most plausible mediators of the association. Fifth, using a one-year follow-up period can be considered a short amount of time for the onset of MDE. Finally, because we only sampled a small number of workers, the findings could not be generalized to the entire population.

## Conclusions

In conclusion, long sitting time at work (9.5+ h per day) might be associated with a high risk of MDE onset among workers. The findings of the present study could motivate employers to reduce the extended sitting time in the workplace to prevent MDD. However, the linear association indicated inverse results. Non-linear associations between sitting time and MDE onset might explain this inconsistency. The evidence for an adverse association between sitting time at work and MDE onset remains inconclusive. Future studies with larger cohorts and greater statistical power should be conducted to confirm this association.

## Data Availability

The datasets used and/or analyzed during the current study are available from the corresponding author upon reasonable request.

## Abbreviations

MDE: major depressive episode
HR: hazard ratio
HDI: highest density interval
RR: relative risk
CI: confidence interval
MDD: major depressive disorders
DSM: Diagnostic and Statistical Manual of Mental Disorders
NHST: null-hypothesis significance testing
CIDI: Composite International Diagnostic Interview
JNIOSH-WLAQ: Worker’s Living Activity-time Questionnaire
GPAQ: Global Physical Activity Questionnaire
METs: metabolic equivalents
MVPA: moderate or vigorous intensity activities
BJSQ: Brief Job Stress Questionnaire
MCMC: Markov chain Monte Carlo
LDEs: light-emitting diodes
